# Late introduction of solids into infants’ diets may increase the risk of food allergy development

**DOI:** 10.1186/s12887-020-02158-x

**Published:** 2020-06-03

**Authors:** Anna Hicke-Roberts, Göran Wennergren, Bill Hesselmar

**Affiliations:** Department of Paediatrics, Sahlgrenska Academy, University of Gothenburg, Gothenburg, Sweden

**Keywords:** Epidemiology, Child, Food allergy, Food intolerance, Risk factors

## Abstract

**Background:**

This study investigated risk factors associated with food allergy or food intolerance among school children in two Swedish towns.

**Methods:**

Questionnaires were used to collect data on self-reported food allergy or intolerance (SRFA) in children aged 7–8 years from Mölndal in southwestern Sweden and Kiruna in northern Sweden. It included questions about specific food allergy or intolerance to cows’ milk, hens’ eggs, fish, peanuts, tree nuts, and cereals and also age of onset, type of symptoms and age of cessation. Information was also gathered on family allergy history, dietary habits, and certain lifestyle aspects.

**Results:**

Of 1838 questionnaires distributed, 1029 were returned: 717/1354 (53%) from Mölndal and 312/484 (64%) from Kiruna. The cumulative incidence of SRFA was 19.6% with a significantly higher cumulative incidence in Kiruna (28.5%) than in Mölndal (15.7%), *P* < .001. Solids were introduced at a later age in Kiruna. Introduction of solids into a child’s diet from the age of 7 months or later, and maternal history of allergic disease, were both risk factors associated with a higher risk of food allergy or intolerance.

**Conclusion:**

Late introduction of solids into an infant’s diet may be one risk factor for developing food allergy or intolerance. Later introduction of solids in Kiruna may be one explanation for the higher cumulative incidence of SRFA in that region.

## Background

Food allergy is an emerging health problem in many countries. It is considered to form part of the “second wave” of allergic diseases, which started decades after the “first wave” comprising asthma, rhino-conjunctivitis, and eczema [1]. Although the increase in the prevalence of first wave allergic diseases like asthma and eczema seems to have levelled off [2–4], the prevalence of food allergy is still increasing [1, 5]. Self-reported food allergy or intolerance is increasingly common today, reported by approximately 15–20% of the children [6, 7], whereas challenge-proven allergy is seen in 3–10% of children in more affluent countries [8–10]. Increasing prevalences are also seen in rapidly developing countries following a changing lifestyle [10–12].

The reason for the increased prevalence of food allergy is still unknown. Genetic factors are important in food allergy, but environmental factors, factors that may also induce epigenetic changes, seem to engender this rapid increase [5, 13]. Identifying modifiable factors may help to prevent or reduce the increasing prevalence. Diet is considered to play an important role. The development of tolerance to food may be influenced by both maternal diet during pregnancy and lactation, as well as by the infant’s diet. Especially the time when complementary food is introduced in the first year of life, and the diversity of the food, has been of interest [13–16].

When to introduce allergenic foodstuff into the child’s diet has been discussed for a long time. Delaying the introduction of allergenic foodstuff such as cow’s milk, hens’ egg and peanuts/tree nuts was recommended especially for high risk children by the American Academy of Pediatrics until the beginning of the twenty first century [17]. But delaying the introduction of allergenic food into the child’s diet has in recent years been questioned, as the previous recommendation to delay the introduction of such foodstuff did not reduce the prevalence of food allergy [14, 18]. Instead, both preclinical and clinical studies indicate that early oral exposures may lead to tolerance [12, 14, 19, 20].

Another dietary aspect in the prevention of allergic diseases is the timing when non-allergenic complementary food should be introduced but so far there are no specific recommendations from an allergy risk perspective [18, 21].

The aim of the study was to investigate risk factors for food allergy development, and analyse the cumulative incidence and symptoms of self-reported food allergy or intolerance (SRFA) among 7- to 8-year-old children in two geographical regions in Sweden, Mölndal in the southwest and Kiruna in the north.

## Methods

### Study design and subjects

A questionnaire was distributed to all school children aged 7–8 years living in two Swedish towns, Mölndal and Kiruna in 2007. Mölndal has a population of 64,000, but it is an integrated part of Gothenburg, a city on the southwest coast of Sweden with a million inhabitants in the urban area. Kiruna is a mining town with 23,000 inhabitants situated north of the Arctic Circle. The questionnaires were distributed and collected by either school nurses or the children’s class teachers to all children aged 7–8 years in all primary schools in both regions. There were no exclusion criteria in the distribution of questionnaire or subject selection. All children were included regardless of the prior history of reported allergies.

The questionnaires were filled in by parents or legal guardians. There was no randomization because all children in this age category were included. The parental report of allergy or intolerance was not confirmed by medical expertise.

### Data collection

The questionnaire focused on asthma and allergic diseases (for an English version, see Additional file 1). It also included questions on the family history of allergic diseases, family and socio-economy, diet and feeding habits. The questions on asthma and allergic diseases (eczema and allergic rhino-conjunctivitis) had been used in two previous cross-sectional studies conducted in the same geographic areas, first in 1979 [22] and second in 1991 [23]. Questions on food introduction and food allergy or food intolerance were added in this study. Questions on feeding habits covered breastfeeding, total duration of breastfeeding, and at what age (in months) different foodstuff was introduced during the child’s first year of life.

The diagnosis of food allergy or food intolerance was based on parent-reported questionnaire replies. A child was labeled as having:

- Food allergy or intolerance if there was a positive answer to the question: “Has your child reacted with allergy or intolerance to any foodstuff?”
- Specific food allergies or intolerances if one or more positive answers were given to the questions: “Has your child reacted with allergy or intolerance to:
  1. milk?
  2. eggs?
  3. fish?
  4. peanuts?
  5. tree nuts or almonds?
  6. cereals?”

Questions about the specific food allergy or intolerance to cows’ milk, hens’ eggs, fish, peanuts, tree nuts, and cereals (ever) included age at onset and possible cessation of symptoms, as well as the type of symptoms. Symptoms were classified as: oral symptoms, diarrhea, rash, edema, respiratory symptoms, vomiting, stomach ache, eczema, urticaria, and rhino-conjunctivitis.

### Data analysis

Data collected from the questionnaires were transferred manually into a Microsoft Access database and double-checked by a second person. IBM SPSS Statistics for Windows (version 22.0.0.0; IBM Corp, Armonk, NY, USA) was used for χ^2^ tests and multiple logistic regression. The significance level was set at 5%.

## Results

A total of 1838 questionnaires were distributed: 1354 in Mölndal and 484 in Kiruna. In Mölndal, 717 were returned compared to 312 in Kiruna, giving response rates of 53 and 64%, respectively. The overall response rate was 56% (1029/1838), with a slightly higher dominance of girls (546/1029, 53%) than boys (483/1029, 47%) but no significant difference in sex ratio was found between the two towns (*P* = 0.168). The questionnaire included 125 questions. Some questions were not answered by all parents or legal guardians. Thus, the number of participants for a question varied in relation to the total number of participants who returned the questionnaires.

The analyses of infant’s diet showed that almost all children (95%) were breastfed, with no difference between the towns (Kiruna 94.8% vs Mölndal 94.6%). Children in Kiruna were, however, breastfed for longer periods than those in Mölndal. The mean duration of any breastfeeding was 10.0 months in Kiruna and 8.7 months in Mölndal (*P* = .004). The difference between towns remained statistically significant when analyzing data from children without a history of milk allergy (10.1 months vs 8.6 months, *P* < 0.0001). The duration of breastfeeding did not differ between children with (9.6 months) vs without (9.1 months) milk allergy (*P* = .594).

Almost all children were introduced to formula or gruel (mixture of cereal and formula) during their first year of life, most commonly, starting at the age of 4–6 months. At this age, significantly more children in Mölndal (*n* = 351/677; 52%) compared to Kiruna (*n* = 125/292; 43%; *P* = .009) started with formula feeds or gruel. Consequently, the opposite was seen in the group aged 7 months or older. In this age group, significantly more children in Kiruna (*n* = 102/292; 35%) than in Mölndal (*n* = 183/677; 27%; *P* = .013) started with formula or gruel.

The majority of children had been introduced to solids during the first 6 months of life (871/988, 88.2%). Solids at this age usually include porridge (mixture of oat and formula) and purees of different fruits, vegetables or root vegetables, but it can also include pasta, rice, meat or fish. Generally, children started with solids earlier in Mölndal than in Kiruna. Solids were started before the age of 4 months in 8.7% (60/693) of the children in Mölndal and in 3.1% (9/295) in Kiruna. Solids were started at the age of 4–6 months in 81% (559/693) of children in Mölndal and in 82% (243/295) in Kiruna, while 11% (74/693) in Mölndal and 15% (43/295) in Kiruna started with solids when the children were 7 months or older (*P* = .002).

The cumulative incidence of SRFA in the children was almost 20%, i.e. one of five children have had immediate- or late-onset symptoms suggestive of food allergy any time during their first 7–8 years of life (Table 1). Of the 201 children with SRFA, 54% were girls. The majority of these children had symptoms related to cow’s milk (12% in the studied population and 60% of those with SRFA) and in slightly more than half of them the symptoms started before three years of age. Allergy to peanuts and tree nuts, hens’ egg, fish and cereals were more uncommon, ranging from 0.7–2.8% in the studied population. Of those with SRFA, 14% reported allergy to peanuts, 13% to hens’ egg, 11% to tree nuts, 5% to fish and 3% to cereals.

**Table 1:** Cumulative incidence of self-reported food allergy or intolerance (SRFA) in children from Mölndal and Kiruna

Children with allergy, n (%)	Total	Mölndal	Kiruna	***P***
No. replying to the question	*n* = 1027	*n* = 715	*n* = 312	
Total (Any SRFA)	201 (19.6)	112 (15.7)	89 (28.5)	<.001
Boys	92 (19)	50 (15.4)	42 (26.6)	.003
Girls	109 (20)	62 (15.9)	47 (30.7)	.001
*P*-value (boys vs girls)	.690	.863	.420	–
No. replying to the question Milk-SRFA	*n* = 1014	*n* = 702	*n* = 312	
Milk total	120 (11.8)	66 (9.4)	54 (17.3)	.001
Early-onset (< 3 years)	74 (7.3)	40 (5.7)	34 (10.9)	.003
Milk-SRFA: skin- manifestation^a^	41 (4.0)	23 (3.3)	18 (5.8)	.062
Milk-SRFA: GI-manifestation^b^	101 (10.0)	55 (7.8)	46 (14.7)	<.001
No. replying to the question Peanut-SRFA	*n* = 999	*n* = 687	*n* = 312	
Total	28 (2.8)	14 (2.0)	14 (4.5)	.039
Peanut-SRFA with symptoms other than only OAS^c^	23 (2.3)	10 (1.5)	13 (4.2)	.008
No. replying to the question Egg-SRFA	*n* = 996	*n* = 686	*n* = 310	
Total	27 (2.7)	16 (2.3)	11 (3.5)	.352
No. replying to the question Tree nut-SRFA	*n* = 999	*n* = 688	*n* = 311	
Total	23 (2.3)	13 (1.9)	10 (3.2)	.222
Tree nut-SRFA with symptoms other than only OAS^c^	14 (1.4)	5 (0.7)	9 (2.9)	.006
No. replying to the question Fish-SRFA	*n* = 995	*n* = 683	*n* = 312	
Total	11 (1.1)	5 (0.7)	6 (1.9)	.159
No. replying to the question Cereal-SRFA	*n* = 995	*n* = 683	*n* = 312	
Total	7 (0.7)	5 (0.7)	2 (0.6)	1.0^d^

^a^Skin-manifestation = symptoms from the skin such as rash, urticaria or eczema, ^b^GI-manifestation = symptoms from the abdomen or gastrointestinal tract such as vomiting, abdominal pain or diarrhea, ^c^OAS = oral allergy syndrome, ^d^Fisher’s exact test

*P* = *P*-value for Mölndal versus Kiruna

There was no significant difference in the cumulative incidence of SRFA when comparing children who received formula or gruel before and after the age of 7 months (*p* = 0.18).

However, there was significant difference in the cumulative incidence of SRFA when comparing the timing of introduction of solids, i.e. when comparing the children who were introduced to solids before the age of 7 months (161/870) and after this age (32/116). Late introduction, from the age of 7 months, resulted in a higher cumulative incidence of SRFA (*p* = 0.001).

Eight putative risk factors for SRFA were analysed with multiple logistic regression (Table 2).

**Table 2:** Multiple logistic regression analysis of cumulative incidence of SRFA in relation to confounding factors

Variable	Adjusted OR	95%CI	***P***
Male	1.179	0.834–1.667	0.35
Duration of breastfeeding	1.014	0.980–1.048	0.429
Father with a history of allergy^a^	1.318	0.932–1.864	0.119
Mother with a history of allergy^a^	1.599	1.126–2.270	0.009
Father’s education, university degree	0.827	0.536–1.276	0.392
Mother’s education, university degree	1.155	0.768–1.737	0.489
Introduction of formula/gruel before 7 months of age	0.715	0.462–1.102	0.129
Late introduction of solids^b^	2.290	1.395–3.761	0.001

^a^ A history of asthma, rhino-conjunctivitis, eczema

^b^ Introduction of solids at 7 months of age or later

Only two of eight independent variables significantly affected the risk, late introduction of solids (OR 2.3) and a mother with a history of allergy (OR 1.6), i.e. late introduction of solids remained an independent risk factor for SRFA. Univariate analyses were used to test several other, less putative, risk factors for food allergy, but we found no statistically significant correlation between SRFA and number of siblings, parental smoking, having animals at home, use of antibiotics, number of respiratory tract infections, or pre-school attendance (data not shown).

To further stress the analyses, we separately investigated if a parental history of food allergy could explain the association between late introduction of solids and SRFA. In a multiple logistic regression, with SRFA as dependent variable and duration of breastfeeding, mother with a history of food allergy, father with a history of food allergy and late introduction of solids as independent variables, late introduction of solids still remained as an independent risk factor for SRFA with OR 1.8 (95% CI 1.15–3.02).

**Fig. 1:**
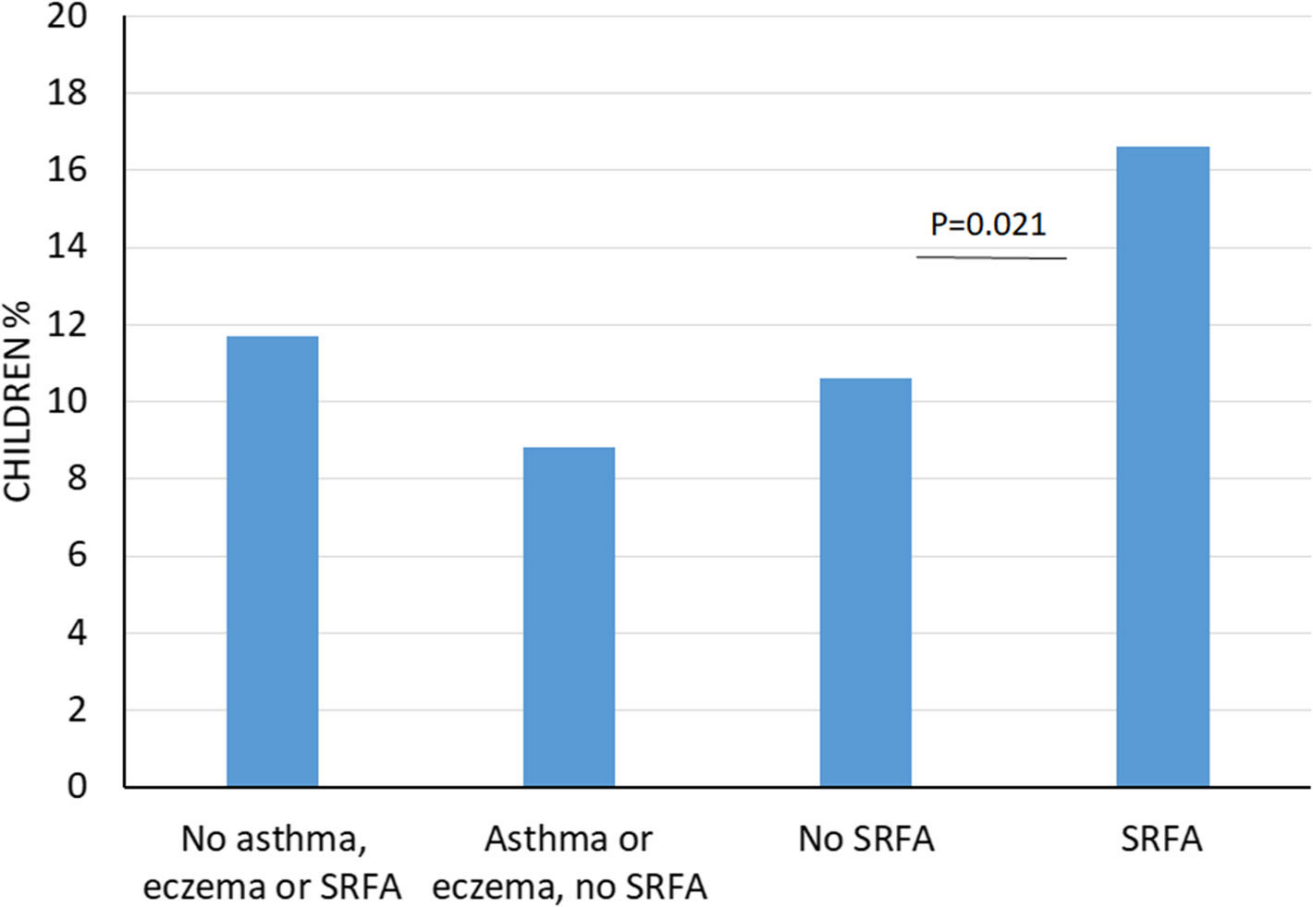
Percentage of children with late introduction of solids and manifestations of allergic disease. Note that while solids often were introduced late in children with SRFA, no such pattern was seen for children with asthma or eczema. SRFA = self reported food allergy or intolerance

In order to evaluate possible reverse causality, that is if families with infants with early signs of allergy may delay introduction of solids, we also made the same analysis for children with asthma or eczema, allergic manifestations that also start early in life, often before 1 year of age. The analysis showed that while solids often were introduced late in children with SRFA, no such pattern was seen for children with asthma or eczema. The results indicate that the late introduction of solids is a risk factor for SRFA and was not a consequence of early allergic symptoms (Fig. 1). To further evaluate if late introduction is causing food allergy, or a consequence of food allergy, we analysed if a similar pattern could be seen in siblings, hypothesising that an association between SRFA in the index child and any allergic disease in siblings could indicate that the parents choose a late introduction because of allergy in siblings, whereas an increased risk of only food allergy in siblings should indicate that late introduction is a consequence of recommendations or habits, and consequently a risk factor for food allergy. The analyses showed that the pattern was similar in the siblings: a late introduction of solids to the index child was associated with a higher ratio of siblings with food allergy (*p* = 0.026), but there was no increased ratio of siblings with asthma (*p* = 0.523) or eczema (*p* = 0.638), supporting the idea that late introduction of solids is a risk factor for SRFA and not a consequence of allergic disease in the index child or in the family (linear regression with duration of breastfeeding and late onset of solids as independent variables).

A history of food allergy (Kiruna: 144/610; 23%, Mölndal: 312/1395; 22%), as well as a history of asthma, ARC or eczema (Kiruna 275/609; 45%, Mölndal 622/1393; 45%) were equally common among parents in Mölndal and Kiruna. Despite this concordance, self-reported food allergy was more common in children in Kiruna than in Mölndal (Table 1). In Kiruna both gruel or formula and solids were overall introduced at a later age as compared to Mölndal, indicating that late introduction of solids to the children in Kiruna may be one explanation to the higher cumulative incidence of self-reported food allergy in Kiruna (28.5%).

**Fig. 2:**
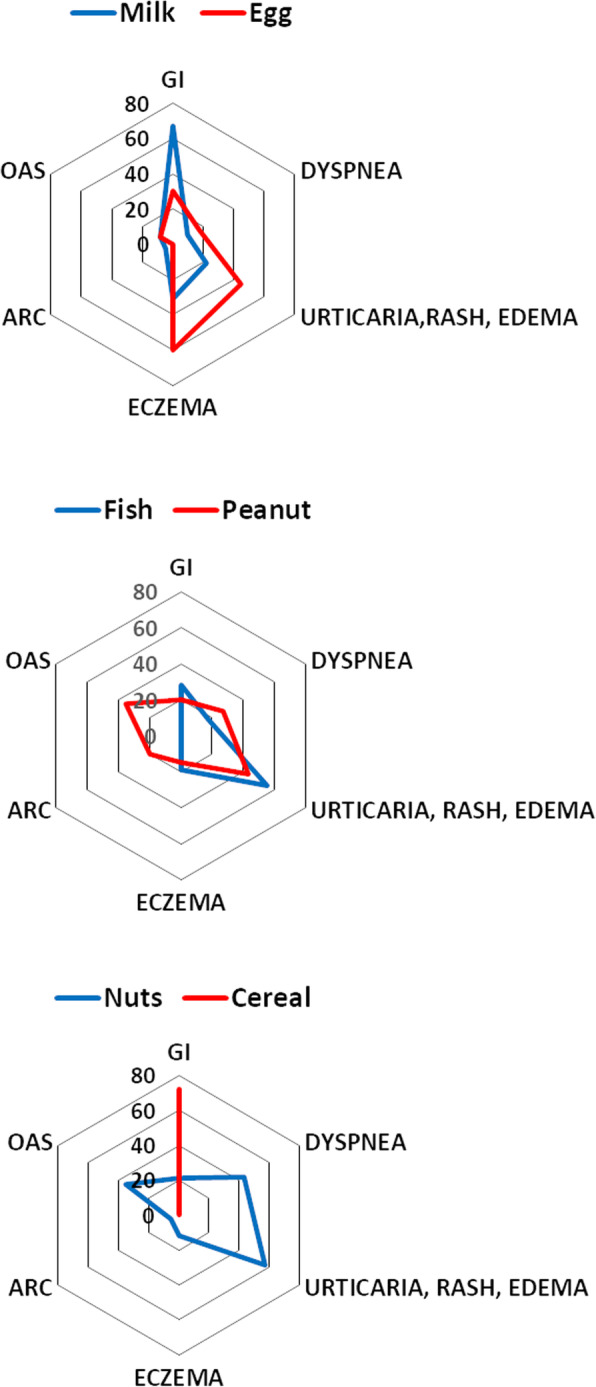
Symptoms in children with self reported food allergy or intolerance. GI = gastro-intestinal symptoms, ARC = allergic rhino-conjunctivitis, OAS = oral allergy syndrome

The symptoms associated with adverse food reactions differed significantly between different food items (Fig. 2). In children with adverse reactions to cow’s milk, three patterns were reported: one with urticaria/rash/angioedema, one with gastrointestinal symptoms, and one with eczema. Urticaria/rash/angioedema reactions are usually associated with IgE sensitisation and immediate-onset, while gastrointestinal symptoms and eczema are usually associated with late-onset and no IgE. Children who reported allergic reactions to hens’ egg usually had skin symptoms with urticaria/rash/angioedema or eczema. Reactions to fish were mainly urticaria/rash/angioedema, whereas reactions to cereals almost always were from the intestine. Reactions to peanuts or tree nuts could include almost any organ system.

## Discussion

The main results of this study were that gruel/formula and solids were introduced later in Kiruna than in Mölndal, and that late introduction of solids was an independent risk factor for SRFA. The late introduction of solids was only associated with a higher cumulative incidence of SRFA, not of asthma or eczema. We speculate that the higher cumulative incidence of SRFA in Kiruna may, at least partly, be explained by a later introduction of solids into the infant’s diet.

Another interesting finding was that a late introduction of solids to the index child was associated with a higher cumulative incidence of SRFA, but not with asthma or eczema, also among siblings. This indicate that late introduction of solids probably is a result of habits or a consequence of recommendations, and not the result of an already existing allergic disease. The absence of correlation between a late introduction of solids and asthma or eczema allows us to believe that there is no reverse causality, since asthma and eczema occur early in life, especially eczema usually presents during the first year of life. Consequently, solids were not introduced at a later age because the child already had asthma or eczema. Neither do we believe that solids were introduced late because of an already existing food allergy since a late introduction of solids remained a risk factor for SRFA also when formula/gruel before the age of 7 months was adjusted for.

Similar to other studies, we also found that a maternal history of allergy and food allergy are risk factors for food allergy among offspring [24]. Differences in parental history of allergy between Kiruna and Mölndal could, consequently, not explain the higher cumulative incidence of SRFA in Kiruna, a suggestion supported by the multiple regression analyses, showing that a late introduction of solids remained an independent risk factor even when parental history of allergic diseases and parental history of food allergy were included as independent variables.

We do not believe that genetic or epigenetic differences can explain our findings since the majority of the population is ethnically similar in both cities, as well is the overall Swedish life style and the fact that the total prevalence of asthma, ARC and eczema is similar in the two regions [4].

The recommendations for infants’ diet have changed several times during the last century. Breastfeeding during the first months of life is undisputable. The recent World Health Organization (WHO) recommendation is 6 months of exclusive breastfeeding, and introduction of complementary feeding from 6 months of age [25]. This is a very important issue in many developing countries with a low prevalence of breastfeeding and a high risk of infections [26]. In industrialized countries, where the risk of life-threatening diarrhea is low, the introduction of complementary food is now recommended between 4 and 6 months of age [27, 28].

The timing of introduction of solids into the diet and its implication on asthma and allergic diseases has been widely discussed during recent decades [29]. The first studies in the twentieth century indicated that an early introduction of solids, before the age of 4 months, may increase the risk of eczema [30] and that avoidance of allergenic food can decrease the risk of allergy among genetically programmed atopic children [31]. The recommendation of postponing the introduction of solids was extended to include all infants [32] and this recommendation was supported by WHO in 2001.

However, more recent studies have shown that late introduction of solids lacks a protective effect on allergy development [33, 34]. Instead, the effect may be the opposite as evidenced by multiple studies, especially the significant KOALA cohort birth study from Southern Netherlands, where delaying the introduction of cow milk products and other food products was associated with higher risk for eczema and recurrent wheeze, the delay introduction of other food products was even associated with higher risk for atopic sensitization [35–37]. Another study from Finland found that late introduction of solid foods was associated with increased risk of allergic sensitization to food and inhalant allergens [36]. Hence, the time of introduction of solids may play an important role in developing immune tolerance [38]. In our study we found that the earlier introduction of solids into infants’ diet may be one of the factors explaining the lower prevalence of food allergy or intolerance in Mölndal as compared to Kiruna.

The mechanism behind development of tolerance to food proteins is complex; it requires a certain state of immune maturation and gut colonization [29]. Exposure to food proteins during a critical early window seems to be an important element in achieving tolerance and this probably occurs between the age of 4 and 7 months [38]. The gut microbiota, its diversity, and its composition play an important role in the development of the human immune system [39, 40] and will change throughout a person’s life span, but most significantly in the early stages of life [41]. Both breastfeeding and weaning are of importance in changing the early gut microbiota pattern [39].

We hypothesize that early introduction of solids may have a dual mechanism: not only presenting food proteins to the child’s immune system during the critical early window, but also allowing them to function as a prebiotic in the establishment of the early gut microbiota pattern. Fruit, vegetables and porridge are usually the first solids introduced to a child. These contain inulin and fructooligosaccharides, which function as a prebiotic (food) for many bacterial groups [39].

There are many studies that concentrate on the early introduction of allergenic foodstuff as egg or peanut [9, 19, 42]. But our study shows that in addition to allergenic food, the timing of complementary food such as porridge, fruit and vegetable purees may be of importance for the development of food allergy or intolerance.

The overall strengths of our study are the uniform methods used in both towns with the same validated questions for asthma, ARC and eczema, targeting the same age groups and similar populations, as well as the overall number of participants in the study [4] and the broad data collection that allowed us to do comparisons between allergies in the index child with allergies in the siblings.

Weaknesses of our study are the retrospective collection of data, that the questions on food allergy were not validated, and that the diagnosis of food allergy was based on self-reported symptoms. This opens up for the possibility of recall bias and overdiagnosis of food allergy. According to studies on the topic, only approximately 10% of reported cases of food allergy/intolerance can be verified by double-blind oral food challenges, which is considered to be the gold standard in the diagnosis of food allergy [29, 43]. Although one can suspect that the prevalence of challenge-proven, current or ongoing, food allergy in these two towns is lower than the reported cumulative incidence of food allergy or intolerance, the differences between the towns and the association between food allergy and late introduction of solids would probably remain the same. Furthermore, even though recall bias may be a problem in cross-sectional studies, it might be similar in both regions. Hence, recall bias is unlikely to influence the difference in cumulative incidence of SRFA between these two regions, neither would it affect the difference between the regions regarding the time when solids were introduced. Reverse causation is another difficult topic to handle in cross-sectional studies. An interventional study method is the only way to handle this issue with hundred percent accuracy, but such studies are difficult to do in healthy infants for several reasons. We have instead used several statistical and methodological methods to analyse possible reverse causality. Even though we believe that the methods we used, solve or minimize the problem, one needs to be aware of the problems with selection bias and reverse causation in all non-interventional studies.

An additional limitation of our study is the response rate, which was approximately 60%, but that level is common in epidemiological studies nowadays. However, we do not suspect a selection bias, as the overall frequency of self-reported food allergy or intolerance is similar to what is presented in other studies [7, 44].

Even though this study supports the idea that early introduction of solids and different foodstuff in the infants diet may induce tolerance, further studies, preferably interventional studies are needed to accurately evaluate a possible protective influence of the early introduction of solids in infant’s diet.

## Conclusions

There was a significant difference in the introduction of formula/gruel and solids between two towns in Sweden, and the cumulative incidence of self-reported food allergy or intolerance was substantially higher in Kiruna than in Mölndal. Later introduction of solids into infants’ diet in Kiruna may be one factor explaining the higher frequency in the northern region.

## Supplementary information

**Additional file 1.** English version of the questionnaire.

**Additional file 2.** Swedish version of the questionnaire.

## Data Availability

The dataset analyzed during the current study are available from the corresponding author on reasonable request.

## Abbreviations

ARC: Allergic rhino-conjunctivitis
CI: Confidence interval
GI: Gastro-intestinal
IgE: Immunoglobulin E
OAS: Oral allergy syndrome
SRFA: Self reported food allergy or intolerance
WHO: World Health Organization
