# Supplementary material for: Late introduction of solids into infants’ diets may increase the risk of food allergy development
Source: BMC Pediatr. 2020 Jun 3;20:273. doi: 10.1186/s12887-020-02158-x (PMC7268275; doi:10.1186/s12887-020-02158-x)
Supplement: Supplementary file 1 — Additional file 1. English version of the questionnaire. [file 12887_2020_2158_MOESM1_ESM.docx]

Questionnaire

Occurrence of asthma and allergy among school children in Sweden and Poland

Date when the questionnaire is answered …………………

1) Name of the child ……………………………………………………………………

Personal identification number …………………………………………………………

Address………………………………………………………………………..

Postal code…………………………… Postal address………………………………

Phone day time ……………………… Mother’s mobile phone ……………………

Phone evening…………………… Father’s mobile phone ……………………

Gender: Girl

Boy

**Informed consent**

I have received and read the copy of the information regarding the study. I have had the right to ask questions regarding the study and my questions have been answered. I am aware that taking part in the study is voluntary and we can withdraw participation of our child at any time during the study without offering a motivation and without influencing our child’s possibility of any future treatment.

Furthermore, I agree to allow any information that is given in the study to be kept in a computer bank.

Date……………………………………….

Parent’s

signature…………………………………………………………………………..

Name in block letters ………………………………………………..

Wheezing module – questions from the ISAAC questionnaire

**Yes No**

2) Has your child ever had wheezing or whistling

in the chest at any time in the past?

IF YOU ANSWERED "NO" PLEASE SKIP TO QUESTION 7

3) Has your child had wheezing or whistling

in the chest in the last 12 months?

IF YOU ANSWERED "NO" PLEASE SKIP TO QUESTION 7

4) How many attacks of wheezing has your child had in the last 12 months?

None

1 to 3

4 to 12

More than 12

5) In the last 12 months, how often, on average, has your child's sleep

been disturbed due to wheezing?

Never woken with wheezing

Less than one night per week

One or more nights per week

6) In the last 12 months, has wheezing ever been severe enough to limit your child's speech to only one or two words at a time between breaths?

7) Has your child ever had asthma?

8) In the last 12 months, has your child's chest sounded wheezy during or after exercise?

9) In the last 12 months, has your child had a dry cough at night,

apart from a cough associated with a cold or a chest infection?

Breathing difficulties – questions based on previous Swedish investigations

**Yes No**

10) Has the child had ***asthma*** or ***wheezing bronchitis***?

If you answered **”NO”,** go directly to question 22

11) At what age were the symptoms first seen? ……………….. year of age

12) At what age did the child last time have the symptoms ?…………… year of age

13) Has the child had asthma or wheezing bronchitis

during the last year?

14) Number of times with symptoms during the last year ……………… times

15) How many days do the symptoms last each time?

1-3 days

4-more

16) Has the child been hospitalised due to asthma or

wheezing bronchitis?

17) If yes, how many times

has the child been hospitalised

due to asthma or

wheezing bronchitis?……………… times

18) When does/did the child get asthma or wheezing bronchitis?

with colds

At exercise

By cold weather

On contact with animals

At leafing (outdoors in May)

Outdoors in June-July

On contact with dust

By foodstuffs

19) Has the child during the ***last 12 months*** needed to stay home from school

due to asthma or wheezing bronchitis?

**Yes No**

20) How many days during the ***last 12 months*** has the child

needed to stay at home from school due to asthma or

wheezing bronchitis?…………………number of days

21) Has the child during the ***last 12 months*** taken any medication

due to asthma or wheezing bronchitis?

If yes, write down what medicines the child takes, what strength and how often the medicine is taken:

| Name of the drug | *Strength* | How often is it taken |
| --- | --- | --- |
|  |  |  |
|  |  |  |
|  |  |  |
|  |  |  |
|  |  |  |
|  |  |  |

## Yes No

## 22) Does the child usually cough during exercise?

## Rhinitis module – questions from the ISAAC questionnaire

##

## Yes No

**23)** Has you child ever had a problem with sneezing, or a runny,

or a blocked nose when he/she DID NOT have a cold or the flu?

IF YOU ANSWERED "NO" PLEASE SKIP TO QUESTION 28

**24)** In the past 12 months, has your child had a problem with

sneezing, or a runny, or a blocked nose when he/she

DID NOT have a cold or the flu?

IF YOU ANSWERED "NO" PLEASE SKIP TO QUESTION 28

25) In the past 12 months, has this nose problem been

accompanied by itchy-watery eyes?

26) In which of the past 12 months did this nose problem occur?

(please tick any which apply)

January  May  September

## February June October

March  July  November

## April August December

27) In the past 12 months, how much did this nose problem interfere

with your child's daily activities?

Not at all

A little

A moderate amount

A lot

28) Has your child ever had hay fever?

**Nose and eye troubles – questions based on previous Swedish investigations**

## Yes No

29) Has the child had ***allergic problems from the nose/eyes?***

If you answered **”NO”,** go directly to question 36

30) At what age were the

troubles first seen?……………… years of age

31) At what age did

the child last have troubles?…………… years of age

32) Has the child had allergic problems from the nose/eyes

during the last year?

33) When does the child get allergic problems from the nose/eyes?

On contact with animals

## At leafing (May)

During June-July

## On contact with dust

34) Has the child during the last year got treatment for

allergic problems from the nose/eyes?

35) If yes, how often?

Occasionally

Spring and/or summer

All-the-year-round

## Eczema module – questions from the ISAAC questionnaire

#### Yes No

36) Has your child ever had an itchy rash which was coming

and going for at least 6 months?

IF YOU ANSWERED "NO" PLEASE SKIP TO QUESTION 42

37) Has your child had this itchy rash at any time

in the last 12 months?

IF YOU ANSWERED "NO" PLEASE SKIP TO QUESTION 42

38) Has this itchy rash at any time affected any of the following places: the folds of the elbows, behind the knees, in front of the ankles, under the buttocks, or around the neck, ears or eyes?

39) At what age did this itchy rash first occur?

Under 2 years

Age 2–4

Age 5 years or more

40) Has this rash cleared completely at any time

during the last 12 months?

41) In the last 12 months, how often, on average, has your child

been kept awake at night by this itchy rash?

Never in the last 12 months

Less than one night per week

One or more nights per week

42) Has your child ever had eczema?

**Skin problems – questions based on previous Swedish investigations**

#### Yes No

43) Has the child had ***eczema***?

If you answered **”NO”,** go directly to question 50

## 44) At what age was

## the eczema first seen?……………….. years of age

45) At what age did the

child last have problems………………….. years of age

46) Has the child had eczema

during the last year?

47) Does the child need daily application of

ointments due to eczema?

48) Which ointments does the child usually use?

Softening ointment/cream Cortisone ointment/cream

Never  Never

Sometimes  Sometimes

Almost every day  Almost every day

Every day  Every day

Name of any other ointment or cream

that the child uses against eczema?…………………………………………….

49) Does the child get an itch from certain foods?

50) Has the child had ***urticaria (hives)/allergic oedema***?

## 51) At what age were the problems

## first seen?……………. years of age

## 52) At what age did the child

## last have problems?………………. years of age

## 53) Has the child had urticaria (hives)/allergic oedema

## during the last year?

## 54) Has the child got a rash from certain food?

**Food allergy/intolerance**

**Yes No**

55) Has the child reacted with allergy or

## intolerance to any foodstuff?

If ”**NO**”, go to question 56

State below for each foodstuff at what age the problems started, at what age they were last seen, and what symptoms/problems the child had.

Milk. The problems started at…….years of age. Problems last time experienced at……years of age.

Symptom/type of problem Itch in the mouth Vomiting

Diarrhoea Stomach pain

Red flush (skin) Eczema

Swollen face Urticaria/hives

Breathing problems Eye/nose troubles

Egg. The problems started at…….years of age. Problems last time experienced at……years of age.

Symptom/type of problem Itch in the mouth Vomiting

Diarrhoea Stomach pain

Red flush (skin) Eczema

Swollen face Urticaria/hives

Breathing problems Eye/nose troubles

Fish. The problems started at…….years of age. Problems last time experienced at……years of age.

Symptom/type of problem Itch in the mouth Vomiting

Diarrhoea Stomach pain

Red flush (skin) Eczema

Swollen face Urticaria/hives

Breathing problems Eye/nose troubles

Peanuts. The problems started at….years of age. Problems last time experienced at……years of age.

Symptom/type of problem Itch in the mouth Vomiting

Diarrhoea Stomach pain

Red flush (skin) Eczema

Swollen face Urticaria/hives

Breathing problems Eye/nose troubles

Other nuts/almonds

The problems started at…….years of age. Problems last time experienced at……years of age.

Symptom/type of problem Itch in the mouth Vomiting

Diarrhoea Stomach pain

Red flush (skin) Eczema

Swollen face Urticaria/hives

Breathing problems Eye/nose troubles

Flour (wheat, oats, barley or rye)

The problems started at…….years of age. Problems last time experienced at……years of age.

Symptom/type of problem Itch in the mouth Vomiting

Diarrhoea Stomach pain

Red flush (skin) Eczema

Swollen face Urticaria/hives

Breathing problems Eye/nose troubles

Other foodstuff (state which)………………………………………………………

The problems started at…….years of age. Problems last time experienced at……years of age.

Symptom/type of problem Itch in the mouth Vomiting

Diarrhoea Stomach pain

Red flush (skin) Eczema

Swollen face Urticaria/hives

Breathing problems Eye/nose troubles

Other foodstuff (state which)………………………………………………………

The problems started at…….years of age. Problems last time experienced

at……years of age.

Symptom/type of problem Itch in the mouth Vomiting

Diarrhoea Stomach pain

Red flush (skin) Eczema

Swollen face Urticaria/hives

Breathing problems Eye/nose troubles

**The child’s father**

#### Yes No

56**)** Has the father had

## *asthma or asthmatic bronchitis?*

If **”NO”,** go directly to question 59

57) At what age were the

problems first seen? ……………… years of age

58) At what age did the

## father last have the problems? ……………….. years of age

## 59) Has the father had *allergic problems from the nose or eyes?*

If **”NO”,** go directly to question 62

60) At what age were the

problems first seen? **…………….** years of age

61) At what age did the

father last have the problems? **……………..** years of age

## 62) Has the father had *eczema?*

If **”NO”,** go directly to question 66

63**)** At what age were the

problems first seen? **……………..** years of age

64) At what age did the

father last have the problems? **……………….** years of age

## 65) Has the eczema been contact allergy?

**Yes No**

66) Has the father had ***urticaria (hives)/allergic oedema****?*

If **”NO”,** go directly to question 69

67**)** At what age were the

problems first seen? **…………….** years of age

68) At what age did the

father last have the problems? **…………………** years of age

####

## 69) Has the father reacted with allergy or

## intolerance to any foodstuff?

## 70) Has the father reacted with allergy or

intolerance to **any foodstuff last year**?

If **”NO”,** go directly to question 72

71) If yes, to which foodstuffs?

Milk  Egg  Fish  Wheat (and other types of flour)

Soy  Apple  Peanuts  Other nuts

Other foodstuffs, state what/which…………………………………………….

……………………………………………………………………………………..

**The child’s mother**

#### Yes No

72) Has the mother had

***asthma or asthmatic bronchitis****?*

If **”NO”,** go directly to question 75

73) At what age were the

problems first seen? ……………… years of age

74) At what age did the

## mother last have the problems? ……………….. years of age

## 75) Has the mother had *allergic problems from the nose or eyes?*

## If ”NO”, go directly to question 78

76) At what age were the

problems first seen? **…………….** years of age

77) At what age did the

mother last have the problems? **……………..** years of age

## 78) Has the mother had *eczema?*

## If ”NO”, go directly to question 82

79**)** At what age were the

problems first seen? **……………..** years of age

80) At what age did the

mother last have the problems? **……………….** years of age

81) Has the eczema been contact allergy?

**Yes No**

82) Has the mother had ***urticaria (hives)/allergic oedema****?*

If **”NO”,** go directly to question 85

83) At what age were the

problems first seen? **…………….** years of age

84) At what age did the

mother last have the problems? **…………………** years of age

####

## 85) Has the mother reacted with allergy or

## intolerance to any foodstuff?

## 86) Has the mother reacted with allergy or

intolerance to **any foodstuff last year**?

If **”NO”,** go directly to question 88

87) If yes, to which foodstuffs?

Milk  Egg  Fish  Wheat (and other types of flour)

Soy  Apple  Peanuts  Other nuts

## Other foodstuffs, state what/which…………………………………………….

……………………………………………………………………………………..

**Questions on family and environment**

**Yes No**

88) In which gestational week was the child born?.............week

89) Was the child born in Sweden?

If ”No”, in which country ……………………….

90) Was the father born in Sweden?

If ”No”, in which country ……………………….

91) Which year was the father born? 19……

92) What is the father’s profession?...................................

93) What is the father’s education? (mark the appropriate box with a cross ”x”)

| Father’s education | | |
| --- | --- | --- |
|  |  | Primary school |
|  |  | Secondary school 2-4 years or corresponding |
|  |  | College or university, max 120 university points |
|  |  | College or university, more than 120 university points |

94) Was the mother born in Sweden?

If ”No”, in which country ……………………….

95) Which year was the mother born? 19……

96) What is the mother’s profession?...................................

97) What is the mother’s education? (mark the appropriate box with a cross ”x”)

| Mother’s education | | |
| --- | --- | --- |
|  |  | Primary school |
|  |  | Secondary school 2-4 years or corresponding |
|  |  | College or university, max 120 university points |
|  |  | College or university, more than 120 university points |

**Yes No**

98) Does the child have siblings?

If ”Yes”, fill in the table below

| **Give the siblings’:** | | | **Do the siblings have:** | | | |
| --- | --- | --- | --- | --- | --- | --- |
| **First name** | **Gender** | **Birth year** | **Asthma** | **Hay fever** | **Eczema** | **Food allergy** |
|  |  |  |  |  |  |  |
|  |  |  |  |  |  |  |
|  |  |  |  |  |  |  |
|  |  |  |  |  |  |  |
|  |  |  |  |  |  |  |
|  |  |  |  |  |  |  |
|  |  |  |  |  |  |  |

99) Did the mother smoke during the pregnancy?

100) Has the father or somebody else in the household smoked

regularly at home when the mother was pregnant with the child?

101) Has anybody smoked at home during the child’s first year?

102) Have you any time during the pregnancy had

dogs, cats or other animals who lived or was mainly kept indoors?

If ”Yes”, state:

Number of dogs…………………..

Number of cats …………………….

Other animals……………………..

103) Have you any time during the child’s first year of life had

dogs, cats or other animals who lived or was mainly kept indoors?

If ”Yes”, state:

Number of dogs…………………..

Number of cats …………………….

Other animals……………………..

**If ”No”, tell why:**

We/I did not want to have animals

Wanted, but could not due to allergy problems in a family member

We/I wanted to avoid the child becoming allergic

Other reason…………………………………………………….

**Yes No**

104) Have you had a dog, cat or another animal

during the last year?

105) Are y of the animals regularly inside at home?

If ”Yes”, state what/which:…………………………….

106) Approximately, how many airway infections (colds)

has the child had?

During the first year of life?

0-5  6-10  more than 10  do not remember

during the last year ?

0-5  6-10  more than 10

107) How many times did the child need to take antibiotics?

During the first year of life ……….. …………………………

In total until now? approximately………………………………..

108) Has the child been at a daycare centre/at a

family day nursery?

If ”**Yes**”:

At what age did the child start at the daycare centre?...........years of age

109) Does the child live together with

both parents  single mother

one parent and a step parent  single father

110) Does the family live in:

detached house, villa semi-detached house, linked house

apartment  farm

Living space………………m^2^

Number of inhabitants………………persons

111) Do you have damp or damage by mold

in the house/apartment?

**The child’s food/diet**

**Yes No**

112) Was the child breastfed?

If ”**Yes**” for how many months?.........................................

113) At what age did the child start to get

formula or gruel? ………..months of age

114) At what age did the child start to get purée

and other food?…………. months of age.

115) Did you use to cook yourself for the child

during the first year of life?

never or almost never

sometimes

approximately half of the time

usually

always

116) How often does the child eat fruit?

never or almost never

1-2 fruits per week

3-6 fruits per week

At least 1 fruit per day

Several fruits per day

117) How often does the child eat vegetables?

never or almost never

1-2 times per week

3-6 times per week

At least once a day

Several times per day

118) How often does the child eat yoghurt?

Never

1-3 times per week

more than 4 times per week

119) How often does the child eat fish?

Never or almost never

1-2 times per month

Once a week

Several times per week

120) Does the child eat food that contains fermented (soured) vegetables

(for example sauerkraut, sour cucumber – not pickled cucumber ) or other food that

is fermented?

Never or almost never

1-2 times per month

Once a week

Several times per week

121) How often do you use semimanufactured food when you cook for the child

(for example fish fingers, meat balls, sausages)?

Never or almost never

1-3 times per month

1-3 times per week

Every day or almost every day

122) How often does the child usually eat ”fast food” (for example hamburgers, pizza)?

Never or almost never

1-3 times per month

1-2 times per week

more than 3 times per week

123) How often does the child usually eat peanuts?

Never

1-2 times per month

1-2 times per week

more than 3 times per week

124) How often does the child usually eat other nuts (for example almonds, hazel nuts etc)?

Never

1-2 times per month

1-2 times per week

more than 3 times per week

125) How often does the child usually drink soft drinks?

Never

1-2 times per month

1-2 times per week

Every day or almost every day

126) How much milk does the child drink?

Never drinks milk

1-2 glassfuls per week

1-2 glassfuls daily

more than 3 glassfuls daily

**Yes No**

127) How often does the child eat game-meat, elk, reindeer, venison,

roe deer or wild boar?

Never or almost never

1-5 times a year

1-2 times a month

once per week or more often

128) Do you sometimes buy egg, meat or unpasteurised milk

directly from the farm?

129) How do you usually wash the dishes?

usually hand dish

usually with dishwasher

Thank you for your participation!

The questionnaire should be given to the school. Please use the attached envelope.
