# Supplementary material for: Late introduction of solids into infants’ diets may increase the risk of food allergy development
Source: BMC Pediatr. 2020 Jun 3;20:273. doi: 10.1186/s12887-020-02158-x (PMC7268275; doi:10.1186/s12887-020-02158-x)
Supplement: Supplementary file 2 — Additional file 2. Swedish version of the questionnaire. [file 12887_2020_2158_MOESM2_ESM.doc]

# Frågeformulär

# Förekomst av astma och allergi bland skolbarn i Sverige och Polen

Datum då formuläret fylls i…………………

1)Barnets namn…………………………………………………………………………

Barnets personnummer……………………………………………………………….

Barnets adress………………………………………………………………………..

Postnummer…………………………… Postadress…………………………………

Tel dagtid ……………………… Mammans mobiltel………………………

Tel kvällstid…………………… Pappans mobiltel…………………………

Kön: Flicka

Pojke

**Informerat samtycke**

Jag har läst och fått en kopia av föräldrainformationen. Jag har haft möjlighet att ställa frågor och mina frågor har besvarats tillfredställande. Jag är medveten om att deltagandet är frivilligt och att vi föräldrar kan avbryta vårt barns medverkan när vi önskar utan närmare motivering, utan att det kommer att påverka vårt barns möjligheter till framtida behandling.

Jag ger också min tillåtelse till att insamlade uppgifter bevaras i dataregister.

Datum……………………………………….

Förälders

Namnteckning…………………………………………………………………………..

Textat namn……………………………………………………………………………..

Andningsbesvär – frågor baserade på ISAAC-formuläret

**Ja Nej**

2) Har Ert barn ***någonsin*** haft väsande eller pipande

andningsljud i bröstet?

Om ni svarat **”NEJ”,**  gå direkt till fråga 7

3) Har Ert barn haft väsande eller pipande andningsljud i bröstet

någon gång under de ***senaste 12 månaderna***?

Om ni svarat **”NEJ”,** gå direkt till fråga 7

4) Hur många episoder med väsande eller pipande andningsljud

i bröstet har Ert barn haft de ***senaste 12 månaderna***?

Ingen

1 – 3 gånger

4 – 12 gånger

Fler än 12 gånger

5) Under de ***senaste 12 månaderna*** hur ofta har i genomsnitt

Ert barns sömn störts av andningsbesvär enligt ovan?

Aldrig vaknat med besvär

Mindre än en natt/vecka

En – flera nätter/vecka

6) Under de ***senaste 12 månaderna*** har Ert barns andningsbesvär

någon gång varit så svår att det endast kunnat säga ett-två ord

mellan andetagen?

7) Har Ert barn ***någonsin*** haft ***astma***?

8) Har Ert barn de ***senaste 12 månaderna*** haft pip i bröstet eller

väsande andning under eller efter ansträngning?

9) Har barnet under de ***senaste 12 månaderna*** haft nattlig

torrhosta utan att ha varit förkyld eller haft en luftrörskattar eller

lunginflammation?

Andningsbesvär – frågor baserade på tidigare Svenska undersökningar

**Ja Nej**

10) Har barnet haft ***astma*** eller ***astmatisk luftrörskatarr***?

Om ni svarat **”NEJ”,** gå direkt till fråga 22

11) Vid vilken ålder märktes

besvären första gången?……………….. års ålder

12) Vid vilken ålder hade

barnet senast besvär?……………………års ålder

13) Har barnet haft astma eller astmatisk luftrörskatarr

senaste året?

14) Antal gånger med

besvär senaste året……………… antal ggr

15) Hur många dagar varar

besvären varje gång?  1-3 dagar

4-fler

16) Har barnet varit inlagt på sjukhus för astma eller

astmatisk luftrörskatarr?

17) Om ja, hur många gånger

har barnet varit inlagt på

sjukhus för astma eller

astmatisk luftrörskatarr?……………… antal ggr

18) När får/fick barnet astma eller astmatisk luftrörskatarr?

Vid förkylning

Vid ansträngning

Av kyla

Vid kontakt med djur

Vid lövsprickning (utomhus i maj)

Utomhusvistelse i juni-juli

Vid kontakt med damm

Av födoämnen

19) Har barnet ***senaste 12 månaderna*** behövt vara hemma från

skolan pga astma eller astmatisk luftrörskatarr?

**Ja Nej**

20) Hur många dagar har

barnet ***senaste 12 månaderna***

behövt vara hemma från

skolan pga astma eller

astmatisk luftrörskatarr?…………………antal dagar

21) Har barnet ***senaste 12 månaderna*** tagit medicin mot

astma eller astmatisk luftrörskatarr?

Om ja, skriv vilka mediciner barnet tar, vilken styrka och hur ofta medicinen tas:

| Medicinens namn | *Styrka* | Hur ofta tas medicinen |
| --- | --- | --- |
|  |  |  |
|  |  |  |
|  |  |  |
|  |  |  |
|  |  |  |
|  |  |  |

## Ja Nej

## 22) Brukar barnet få hosta vid ansträngning?

## Näs- och ögonbesvär - frågor baserade på ISAAC formuläret

##

## Ja Nej

## 23) Har Ert barn *någonsin* varit besvärad av nysningar,

rinnsnuva eller nästäppa utan att ha varit förkylt?

Om ni svarat **”NEJ”,** gå direkt till fråga 28

## 24) Har Ert barn under de *senaste 12 månaderna* varit

## besvärad av nysningar, rinnsnuva eller nästäppa

utan att ha varit förkylt?

Om ni svarat **”NEJ”,** gå direkt till fråga 28

25) Har under de ***senaste 12 månaderna*** dessa näsbesvär

åtföljts av kliande, rinnande ögon?

26) I vilken/vilka månader hade Ert barn näsbesvär

(sätt ”x” i lämpliga rutor)?

januari  maj  september

## februari juni oktober

mars  juli  november

## april augusti december

27 Under de ***senaste 12 månaderna***, hur mycket påverkade

näsbesvären Ert barns dagliga aktiviteter?

Inte alls

Något lite

Måttligt

Ganska mycket

28) Har Ert barn ***någonsin*** haft ”hösnuva”?

**Näs- och ögonbesvär – frågor baserade på tidigare Svenska undersökningar**

## Ja Nej

29) Har barnet haft ***allergisk snuva/ögonkatarr?***

Om ni svarat **”NEJ”,** gå direkt till fråga 36

30) Vid vilken ålder märktes

besvären första gången?……………… års ålder

31) Vid vilken ålder hade

barnet senast besvär………………….. års ålder

32) Har barnet haft allergisk snuva/ögonkatarr

senaste året?

33) När får barnet allergisk snuva/ögonkatarr?

Vid kontakt med djur

## Vid lövsprickning (maj)

Under juni-juli månad

## Vid kontakt med damm

34) Har barnet senaste året fått behandling för

allergisk snuva/ögonkatarr?

35) Om ja, hur ofta?

Enstaka gånger

Vår och/eller sommar

Året runt

## Hudbesvär – frågor baserade på ISAAC-formuläret

#### Ja Nej

36) Har Ert barn ***någonsin*** haft ett kliande utslag som

kommit och gått under minst 6 månader?

Om ni svarat **”NEJ”,** gå direkt till fråga 42

## 37) Har Ert barn haft detta kliande utslag någon

## gång under de senaste 12 månaderna?

Om ni svarat **”NEJ”,** gå direkt till fråga 42

38) Har detta kliande utslag vid något tillfälle förekommit

på något av följande ställen: *armveck, knäveck, fotleder,*

*på lårens baksidor eller på halsen, kring ögonen eller öronen*?

39) Vid vilken ålder sågs utslagen första gången?

Före 2 års ålder

mellan 2 och 5 års ålder

Från fyllda 5 års ålder

40) Har detta utslag försvunnit helt vid något tillfälle

under de ***senaste 12 månaderna***?

41) Under de **senaste 12 månaderna**, hur ofta, i genomsnitt,

har det kliande utslaget hållit Ert barn vaket nattetid?

Aldrig

Inte så ofta som en natt/vecka

En eller flera nätter/vecka

42) Har Ert barn ***någonsin*** haft eksem?

**Hudbesvär – frågor baserade på tidigare Svenska undersökningar**

#### Ja Nej

43) Har barnet haft ***eksem***?

Om ni svarat **”NEJ”,** gå direkt till fråga 50

## 44) Vid vilken ålder märktes

## eksemet första gången?……………….. års ålder

45) Vid vilken ålder hade

barnet senast besvär………………….. års ålder

46) Har barnet haft eksem

senaste året?

47) Behöver barnet daglig smörjning pga

eksem?

48) Vilka salvor brukar barnet använda?

Mjukgörande salva/kräm Kortisonsalva/kräm

Aldrig  Aldrig

Ibland  Ibland

Nästan varje dag  Nästan varje dag

Varje dag  Varje dag

Ange namn på annan salva eller kräm

som barnet använder mot eksem?…………………………………………….

49) Får barnet klåda av viss mat?

50) Har barnet haft ***nässelutslag/allergisk svullnad***?

## 51) Vid vilken ålder märktes

## besvären första gången?……………. års ålder

## 52) Vid vilken ålder hade

## barnet senast besvär?………………. års ålder

## 53) Har barnet haft nässelutslag/allergisk

## svullnad senaste året?

## 54) Har barnet fått utslagen av viss mat?

**Födoämneallergi/överkänslighet**

**Ja Nej**

55) Har barnet reagerat med allergi eller

## överkänslighet mot något födoämne?

Om ”**NEJ**”, gå till fråga 56

Ange nedan för varje födoämne vid vilken ålder besvären började, vid vilken ålder de märktes senast, och vilka symtom/besvär barnet hade

Mjölk besvären började vid…….års ålder Besvär senast vid……års ålder

Symtom/typ av besvär Klåda i munnen Kräkningar

Diarré Magvärk

Hudrodnad Eksem

Svullnad i ansikte Nässelutslag

Andningsbesvär Ögon/näsbesvär

Ägg besvären började vid……. års ålder Besvär senast vid……års ålder

Symtom/typ av besvär Klåda i munnen Kräkningar

Diarré Magvärk

Hudrodnad Eksem

Svullnad i ansikte Nässelutslag

Andningsbesvär Ögon/näsbesvär

Fisk besvären började vid……. års ålder Besvär senast vid……års ålder

Symtom/typ av besvär Klåda i munnen Kräkningar

Diarré Magvärk

Hudrodnad Eksem

Svullnad i ansikte Nässelutslag

Andningsbesvär Ögon/näsbesvär

Jordnötter besvären började vid……. års ålder Besvär senast vid……års ålder

Symtom/typ av besvär Klåda i munnen Kräkningar

Diarré Magvärk

Hudrodnad Eksem

Svullnad i ansikte Nässelutslag

Andningsbesvär Ögon/näsbesvär

Andra nötter/mandel

besvären började vid……. års ålder Besvär senast vid……års ålder

Symtom/typ av besvär Klåda i munnen Kräkningar

Diarré Magvärk

Hudrodnad Eksem

Svullnad i ansikte Nässelutslag

Andningsbesvär Ögon/näsbesvär

Mjöl (vete, havre, korn eller råg)

besvären började vid…….års ålder Besvär senast vid……års ålder

Symtom/typ av besvär Klåda i munnen Kräkningar

Diarré Magvärk

Hudrodnad Eksem

Svullnad i ansikte Nässelutslag

Andningsbesvär Ögon/näsbesvär

annat födoämne (ange vilket)………………………………………………………

besvären började vid…….års ålder Besvär senast vid……års ålder

Symtom/typ av besvär Klåda i munnen Kräkningar

Diarré Magvärk

Hudrodnad Eksem

Svullnad i ansikte Nässelutslag

Andningsbesvär Ögon/näsbesvär

annat födoämne (ange vilket)………………………………………………………

besvären började vid…….års ålder Besvär senast vid……års ålder

Symtom/typ av besvär Klåda i munnen Kräkningar

Diarré Magvärk

Hudrodnad Eksem

Svullnad i ansikte Nässelutslag

Andningsbesvär Ögon/näsbesvär

**Barnets pappa**

#### Ja Nej

56**)** Har pappan haft

## *astma eller astmatisk luftrörskatarr?*

Om **”NEJ”,** gå direkt till fråga 59

57) Vid vilken ålder märktes

besvären första gången? ……………… års ålder

58) Vid vilken ålder hade

## pappan senast besvär? ……………….. års ålder

## 59) Har pappan haft *allergisk snuva/ögonkatarr?*

Om **”NEJ”,** gå direkt till fråga 62

60) Vid vilken ålder märktes

## besvären första gången? ……………. års ålder

61)Vid vilken ålder hade

## pappan senast besvär? …………….. års ålder

## 62) Har pappan haft *eksem?*

Om **”NEJ”,** gå direkt till fråga 66

63**)** Vid vilken ålder märktes

## besvären första gången? …………….. års ålder

64)Vid vilken ålder hade

## pappan senast besvär? ………………. års ålder

## 65) Har eksemet varit kontaktallergi?

**Ja Nej**

66) Har pappan haft ***nässelutslag/allergisk svullnad****?*

Om **”NEJ”,** gå direkt till fråga 69

67**)** Vid vilken ålder märktes

## besvären första gången? ……………. års ålder

68) Vid vilken ålder hade

## pappan senast besvär? ………………… års ålder

####

## 69) Har pappan reagerat med allergi eller

## överkänslighet mot något födoämne?

70) Har pappan reagerat med allergi eller

överkänslighet mot **något födoämne sista året**?

Om **”NEJ”,** gå direkt till fråga 72

71) Om ja, mot vilka födoämnen?

Mjölk  Ägg  Fisk  Vete (och andra mjölsorter)

Soja  Äpple  Jordnöt  Andra nötter

Annat födoämne, ange vilket/vilka…………………………………………….

……………………………………………………………………………………..

**Barnets mamma**

#### Ja Nej

72) Har mamman haft

## *astma eller astmatisk luftrörskatarr?*

Om **”NEJ”,** gå direkt till fråga 75

73) Vid vilken ålder märktes

besvären första gången? ……………… års ålder

74) Vid vilken ålder hade

## mamman senast besvär? ……………….. års ålder

## 75) Har mamman haft *allergisk snuva/ögonkatarr?*

Om **”NEJ”,** gå direkt till fråga 78

76) Vid vilken ålder märktes

## besvären första gången? ……………. års ålder

77)Vid vilken ålder hade

## mamman senast besvär? …………….. års ålder

## 78) Har mamman haft *eksem?*

Om **”NEJ”,** gå direkt till fråga 82

79**)** Vid vilken ålder märktes

## besvären första gången? …………….. års ålder

80)Vid vilken ålder hade

## mamman senast besvär? ………………. års ålder

## 81) Har eksemet varit kontaktallergi?

#### Ja Nej

82) Har mamman haft

***nässelutslag/allergisk svullnad?****?*

Om **”NEJ”,** gå direkt till fråga 85

83) Vid vilken ålder märktes

## besvären första gången? ……………. års ålder

84) Vid vilken ålder hade

## mamman senast besvär? …………………års ålder

####

## 85) Har mamman reagerat med allergi eller

## överkänslighet mot något födoämne?

86) Har mamman reagerat med allergi eller

överkänslighet mot **något födoämne sista året**?

Om **”NEJ”,** gå direkt till fråga 88

87) Om ja, mot vilka födoämnen?

Mjölk  Ägg  Fisk  Vete (och andra mjölsorter)

Soja  Äpple  Jordnöt  Andra nötter

Annat födoämne, ange vilket/vilka…………………………………………….

……………………………………………………………………………………..

**Frågor om familj och miljö**

**JA Nej**

88) I vilken graviditetsveckan föddes barnet?.............vecka

89) Föddes barnet i Sverige?

Om ”Nej” i vilken land ……………………….

90) Föddes pappan i Sverige?

Om ”Nej” i vilken land ……………………….

91) Vilket år föddes pappa? 19……

92)Vilket yrke ha pappa ?...................................

93)Vilken utbildning har pappa?(sätta en kryss i motsvarande rutan)

| Pappans utbildning | | |
| --- | --- | --- |
|  |  | Grundskola |
|  |  | Gymnasium 2-4år el motsvarande |
|  |  | Högskola eller universitet, max 120 poäng |
|  |  | Högskola el universitet > 120 poäng |

94) Föddes mamma i Sverige?

Om ”Nej” i vilken land ……………………….

95) Vilket år föddes mamma? 19……

96) Vilket yrke har mamma?.............................

97) Vilken utbildning har mamma? (sätta en krys i motsvarande rutan)

| Mammans utbildning | | |
| --- | --- | --- |
|  |  | Grundskola |
|  |  | Gymnasium 2-4år el motsvarande |
|  |  | Högskola eller universitet, max 120 poäng |
|  |  | Högskola el universitet > 120 poäng |

**JA Nej**

98) Har barnet syskon?

Om ”Ja”, fyll i tabellen nedan

| **Ange syskonens:** | | | **Har syskonen:** | | | |
| --- | --- | --- | --- | --- | --- | --- |
| **Förnamn** | **kön** | **födelseår** | **astma** | **hösnuva** | **eksem** | **Födoämnesallergi** |
|  |  |  |  |  |  |  |
|  |  |  |  |  |  |  |
|  |  |  |  |  |  |  |
|  |  |  |  |  |  |  |
|  |  |  |  |  |  |  |
|  |  |  |  |  |  |  |
|  |  |  |  |  |  |  |

99) Rökte mamman under graviditeten?

100) Har pappan eller någon annan i hushållet rökt regelbundet

inomhus medan mamman var gravid med barnet?

101) Har någon rökt inomhus under barnets första levnadsår?

102) Har Ni någon gång under graviditeten haft

hundar, katter eller andra djur som bott eller i huvudsak

vistats inomhus?

Om ”Ja”, ange:

Antal hundar:…………………..

Antal katter…………………….

Andra djur……………………..

103) Hade Ni någon gång under barnets första levnadsår

hundar, katter eller andra djur som bott eller i huvudsak

vistats inomhus?

**Om ”Ja”, ange:**

Antal hundar:…………………..

Antal katter …………………….

Andra djur……………………..

**Om ”Nej”, ange varför:**

vi/jag ville inte ha djur

ville, men kunde ej pga allergibesvär hos någon i familjen

vi/jag ville undvika att barnet blev allergiskt

annat skäl…………………………………………………….

**Ja Nej**

104)Har ni haft hund, katt eller annat djur

under senaste året?

105)Vistas något av djuren regelbundet inomhus?

Om ”Ja”, ange vilket/vilka:…………………………….

106) Ungefär hur många luftvägsinfektioner (förkylningar)

har barnet haft?

Under första levnadsåret?

0-5  6-10  mer än 10  minns ej

under senaste året ?

0-5  6-10  mer än 10

107) Hur många gånger behövde barnet ta antibiotika?

Under det första levnadsåret……….. …………………………

Totalt under hela livet? ungefär………………………………..

108) Har barnet varit på daghem/hos dagmamma?

Om ”**JA**”:

I vilket ålder började barnet på daghem?...........års ålder

109)Bor barnet tillsammans med

båda föräldrarna  ensamstående mamma

en förälder och styvförälder  ensamstående pappa

110) Bor familjen i:

villa rad- eller kedjehus

lägenhet  bondgård

Bostad storlek………………m2

Antal boende………………personer

111)Har Ni fukt eller mögelskada i bostaden?

**Barnets kost**

**Ja Nej**

112)Ammades barnet?

Om ”**JA**” hur många månader?.........................................

113) Vid vilken ålder började barnet få

tillägg eller välling ? ………..månaders ålder

114) Vid vilken ålder började barnet få pure

och annan mat?………….månaders ålder?

115) Brukade ni lagat mat själv till barnet

under första levnadsåret?

aldrig eller nästan aldrig

ibland

ung. hälften

oftast

alltid

116) Hur ofta äter barnet frukt?

aldrig eller nästan aldrig

1-2 frukter per vecka

3-6 frukter per vecka

Minst 1 frukt per dag

Flera frukter per dag

117) Hur ofta äter barnet grönsaker?

aldrig eller nästan aldrig

1-2 ggr per vecka

3-6 ggr per vecka

Minst 1 gång per dag

Flera ggr per dag

118) Hur ofta äter barnet yoghurt?

Aldrig

1-3 ggr per vecka

mer än 4 ggr per vecka

119) Hur ofta äter barnet fisk?

Aldrig eller nästan aldrig

1 -2 ggr per månad

1 ggr per vecka

flera ggr per vecka

120) Äter barnet mat som innehåller fermenterade (surgjorda) grönsaker

(t.ex. surkål, sur gurka – inte inlagd gurka ) eller annat mat som

är fermenterad?

Aldrig eller nästan aldrig

1 -2 ggr per månad

1 ggr per vecka

flera ggr per vecka

121) Hur ofta brukar Ni använda halvfabrikat när Ni lagar mat åt barnet

(t.ex. fiskpinnar, köttbullar, korv)?

Aldrig eller nästan aldrig

1-3 ggr per månad

1-3 ggr per vecka

varje dag eller nästan varje dag

122) Hur ofta brukar barnet äta ”snabbmat ” (t.ex. hamburgare, pizza)?

Aldrig eller nästan aldrig

1-3 ggr per månad

1-2 ggr per vecka

mer än 3 ggr per vecka

123) Hur ofta brukar barnet äta jordnötter?

Aldrig

1-2 ggr per månad

1-2 ggr per vecka

mer än 3 ggr per vecka

124) Hur ofta brukar barnet äta andra nötter (tex mandel, hasselnöt etc)?

Aldrig

1-2 ggr per månad

1-2 ggr per vecka

mer än 3 ggr per vecka

125) Hur ofta brukar barnet dricka läsk?

Aldrig

1-2 ggr per månad

1-2 ggr per vecka

varje dag eller nästan varje dag

126) Hur mycket mjölk dricker barnet?

Aldrig

1-2 glas per vecka

1-2 glas dagligen

mer än 3 glas dagligen

**Ja Nej**

127)Hur ofta äter barnet viltkött, älg, ren, hjort, rådjur eller

vildsvin?

Aldrig eller nästan aldrig

1-5 ggr per år

1-2 ggr per månad

en gång per vecka eller oftare

128)Köper ni ibland ägg, kött eller opastöriserad mjölk

direkt från bondgården?

129) Hur brukar ni diska?

oftast handdisk

oftast i diskmaskin

Tack för er medverkan

Frågeformuläret lämnas till skolan. Använd gärna bifogat kuvert.
